# Respiratory Syncytial Virus Persistence in Murine Macrophages Impairs IFN-β Response but Not Synthesis

**DOI:** 10.3390/v7102879

**Published:** 2015-10-16

**Authors:** Evelyn Rivera-Toledo, Laura Torres-González, Beatriz Gómez

**Affiliations:** Department of Microbiology and Parasitology, Faculty of Medicine, National Autonomous University of Mexico (UNAM), Ciudad Universitaria, D.F. Mexico C.P. 04510, Mexico; evelynmicro@gmail.com (E.R.-T.); tgonzalezlaura@gmail.com (L.T.-G.)

**Keywords:** respiratory syncytial virus, viral persistence, IFN-beta, STAT1, IRF3

## Abstract

Type-I interferon (IFN-I) production is an early response to viral infection and pathogenic viruses have evolved multiple strategies to evade this cellular defense. Some viruses can establish and maintain persistent infections by altering the IFN-I signaling pathway. Here, we studied IFN-I synthesis and response in an *in vitro* model of persistent infection by respiratory syncytial virus (RSV) in a murine macrophage-like cell line. In this model, interferon regulatory factor 3 was constitutively active and located at nuclei of persistently infected cells, inducing expression of IFN-beta mRNA and protein. However, persistently infected macrophages did not respond in an autocrine manner to the secreted-IFN-beta or to recombinant-IFN-beta, since phosphorylated-STAT1 was not detected by western blot and transcription of the interferon-stimulated genes (ISGs) *Mx1* and *ISG56* was not induced. Treatment of non-infected macrophages with supernatants from persistently infected cells induced STAT1 phosphorylation and ISGs expression, mediated by the IFN-I present in the supernatants, because blocking the IFN-I receptor inhibited STAT1 phosphorylation. Results suggest that the lack of autocrine response to IFN-I by the host cell may be one mechanism for maintenance of RSV persistence. Furthermore, STAT1 phosphorylation and ISGs expression induced in non-infected cells by supernatants from persistently infected macrophages suggest that RSV persistence may trigger a proinflammatory phenotype in non-infected cells as part of the pathogenesis of RSV infection.

## 1. Introduction

Respiratory syncytial virus (RSV), a negative-sense, single-stranded RNA virus of the family Paraxmyxoviridae (genus *Pneumovirus*), is considered the main cause of bronchiolitis and pneumonia in children under two years of age [1,2,3]. Most infants experience RSV infection during the first year of life [4,5] and after severe RSV disease, 30%–40% of this affected population will undergo recurrent wheezing or asthma-like symptoms in later childhood [6,7,8]. The mechanisms underlying long-term airway hyperactivity are incompletely understood, but may be explained in part by a low-level, persistent viral infection, which may cause a chronically stimulated immune response [7,9]. RSV persistence in humans, although not confirmed, has been suggested by studies that reported the isolation of RSV from the nasopharynx of apparently healthy children [10], the presence of genomic RSV-RNA in respiratory-tract secretions from adult patients with stable chronic obstructive pulmonary disease [11], and the detection of viral mRNA in post-mortem lung tissue from infants who died without apparent clinical disease [12]. Moreover, the RSV genome has been detected in primary human bone-marrow stromal cells from adult and child donors [13], suggesting that RSV may establish persistent infections in respiratory and non-respiratory tissues. To study the mechanisms and consequences of persistent viral infections, cell cultures and animal models are useful tools; RSV persistence has been established in both types of models [14,15,16,17,18].

Interferon regulatory factor 3 (IRF3) is a transcription factor that mediates type-I interferon (IFN-I) expression, such as IFN-α and IFN-β, after viral infection [19,20]. As IFN-I activity is an efficient mechanism to control viral replication, many pathogenic viruses have developed strategies to overcome IFN-I expression in order to guarantee their replication and spread. IRF3 inactivation, achieved through several viral strategies, has been reported to be associated with persistent infections [21,22,23].

Triggering of the IFN-I signaling pathway during viral replication implies recognition of viral 5′-triphosphate-RNA and double-stranded RNA intermediates by the cytosolic, retinoic acid-inducible gene I (RIG-I)-like receptors (RLRs) of the innate immune system, such as RIG-I and MDA5 [24]. Upon ligand binding, RLRs form homo-dimers that migrate to the outer membrane of mitochondria, where a macromolecular signaling complex is assembled [25,26]. Consequently, IRF3 undergoes phosphorylation, dimerization, and nuclear translocation where along with the transcription factors NF-kB and AP-1, it binds the IFN-β promoter to induce gene expression [27,28]. Secreted IFN-β acts in an autocrine/paracrine manner via the IFNα/β receptor (IFNAR), eliciting phosphorylation of STAT2 and STAT1 through the Jak and Tyk2 kinases. Phosphorylated-STAT1 (p-STAT1), with STAT2 and IRF9, forms the interferon-stimulated gene factor-3 complex, which is translocated to the nucleus, thereby inducing the expression of a variety of interferon-stimulated genes (ISGs), including myxovirus-resistance gene 1 (*Mx1*) and interferon-induced protein with tetratricopeptide repeats 1 (*IFIT1/ISG56*), to create an antiviral state [29,30].

Acute RSV infection of epithelial cell lines and of primary macrophages induces a relatively low production of IFN-α and -β. Synthesis of IFN-α and -β is significantly augmented, when infection is achieved with mutant RSV that lack the non-structural viral genes 1 and 2 (NS1 and NS2) [31]. RSV NS1 and NS2 proteins are responsible for disrupting cellular IFN-I signaling at the levels of expression and response by acting on multiple targets, such as IRF-3 and STAT2, respectively [32,33,34,35,36].

Whereas most studies that were focused on determining the mechanisms by which RSV evades the IFN-I response have been performed during acute infections, we studied the state of activation of the IFN-I signaling pathway at the level of synthesis and response during persistent RSV infection by using a previously established *in vitro* model of persistently RSV-infected murine macrophages (MΦP) [14]. Here, we report that MΦP constitutively expressed the active form of IRF3 and consequently IFN-β, but these macrophages were not competent to respond to this cytokine in an autocrine manner, as phosphorylation of STAT1 was not detected, neither expression of the ISGs *Mx1* nor *ISG56*. However, IFN-β secreted by MΦP was able to activate STAT1 in non-infected macrophages along with *Mx1* and *ISG56* mRNA expression, thereby suggesting that persistent RSV infection may not only affect the host cell, but also indirectly affect surrounding cells by altering their state of activation, contributing with the pathogenesis of RSV infection.

## 2. Materials and Methods

### 2.1. Cell Lines and Virus

The MΦP culture was obtained in our laboratory as previously reported [14]. Briefly, the murine macrophage-like cell line P388D1, obtained from the American Type culture Collection (ATCC, TIB 63; Rockville, MD, USA), was infected at a multiplicity of infection (m.o.i.) of 1 by using the RSV wild-type strain Long (ATCC, VR-26) (wtRSV). Surviving cells were subcultured for ten passages and then were superinfected (m.o.i. of 1), resulting in 90%–95% of cells expressing viral antigens. After ten passages of that superinfected culture, neither cellular destruction nor syncytia formation were observed, although more than 90% of cells expressed viral antigens and the supernatants had low levels of infectious viruses (1–3 × 10^2^ 50% tissue culture infective dose (TCID_50_)/mL) [14]. In parallel to MΦP, the original P388D1 cell line was propagated as a non-infected control cell line (MΦN) in supplemented-RPMI (RPMI 1640 cell-culture medium (GIBCO/BRL, Grand Island, NY, USA), containing 5% fetal bovine serum (Biowest, Veracruz, Mexico), 1% penicillin-streptomycin (Invitro S.A., Mexico City), and 1 μM 2-mercaptoethanol) (37 °C; 5% CO_2_).

The human epithelial cell line Hep-2, was cultured in Dulbecco’s Modified Eagle Medium (GIBCO/BRL, Grand Island, NY, USA), supplemented with 5% fetal bovine serum, 10 mM HEPES (SIGMA, St. Louis, MO, USA), and 1% penicillin-streptomycin. Hep-2 cells were used to propagate the wtRSV and to determine viral infectivity, expressed in TCID_50_/mL [37]. The wtRSV obtained in this manner was used to infect cultures of P388D1 macrophages (m.o.i. of 2) for 6, 24, and 48 h, in order to evaluate the expression of p-IRF3 in these acutely RSV-infected macrophages (MΦA). Under such conditions, we observed by direct immunofluorescence 65%–75% and 85%–90% of infected cells at 24 and 48 h post-infection (p.i.), respectively. The percentage of acutely infected cells at 48 h p.i., was similar to the percentage of persistently infected macrophages in the MΦP cultures.

### 2.2. Confirmation of Persistent RSV Infection in MΦP

In this study, MΦP from passages 85–105 were used to perform the experiments. Throughout the passages, RSV persistence was monitored (every two or three weeks) by immunofluorescence to determine the expression of viral antigens and by conventional RT-PCR to detect mRNA of the nucleocapsid (*N*) gene. Direct immunofluorescence was performed with a FITC-labeled, polyclonal antibody anti-RSV directed against the N and F viral proteins (Oxoid, Hampshire, UK). Briefly, 0.5 × 10^6^ MΦP or MΦN were fixed with 4% p-formaldehyde (15 min) and permeabilized with 0.3% saponine (15 min). After non-specific binding sites were blocked by incubating cells (25 min; on ice), with blocking solution (PBS containing 5% neonate bovine serum (Bioexport, Mexico)), a dilution of polyclonal anti-RSV (1:10 in blocking solution) was added to the cells and the mixture was incubated (35 min; on ice). The percentage of FITC-positive cells, as determined by flow cytometry (FACScan; Becton Dickinson, Mountain View, CA, USA) was reported. The expression of *N* gene mRNA was analyzed by using a primer pair to amplify a 1187-bp segment; as a control, mRNA of the *GAPDH* gene was determined with a primer pair to amplify a 260-bp segment, as previously described [38].

### 2.3. Western Blot

Whole-cell extracts were prepared from MΦN and MΦP, and where indicated, from MΦA. Ice-cold lysing buffer (50 mM Tris-HCl, pH 8.0, 150 mM NaCl, 1% Triton X-100, 1% sodium deoxycholate, and 0.1% SDS), containing EDTA-free complete protease inhibitor cocktail (Roche, Indianapolis, IN, USA), was added to 3–5 × 10^6^ cells. After incubation (15 min; on ice), lysates were centrifuged (13,000 rpm) and supernatants collected. The concentration of total protein in each sample was determined by using Bradford reagent (Bio-Rad, Hercules, CA, USA). Proteins (30 μg) were separated by SDS-polyacrylamide gel electrophoresis on a NuPAGE bis-Tris gradient gel with 4%–12% acrylamide (Invitrogen, Carlsbad, CA, USA) and transferred to polyvinylidene difluoride membranes (Amersham, Piscataway, NJ, USA). Specific proteins were analyzed with the primary monoclonal antibodies, anti-p-IRF3 or anti-p-STAT1 (Cell Signaling, Beverly, MA, USA), diluted 1:1000 in TBS-T buffer with 5% BSA. Monoclonal anti-STAT1 was diluted 1:500, while polyclonal anti-IRF3 and anti-β-tubulin (Santa Cruz Biotechnology, CA, USA) were diluted at 1:1000 in TBS-T buffer containing 5% non-fat milk. All membranes were incubated with primary antibody (overnight; 4 °C), washed three times with TBS-T and incubated with the appropriate peroxidase-conjugated secondary antibody, diluted 1:3000 in TBS-T containing 5% non-fat milk (2 h; room temperature).

Western blots were analyzed by chemiluminescence with the SuperSignal kit (Pierce Biotechnology, Rockford, IL, USA). Densitometry analysis of the resulting bands was performed with images obtained with the ChemiDoc™ XRS (Bio-Rad) and analyzed by using QuantityOne software (Bio-Rad).

### 2.4. Subcellular Fractionation

Cell pellets with 5 × 10^6^ MΦN or MΦP were resuspended in a cold cytoplasmic-lysing buffer (10 mM HEPES pH 7.9, 1.5 mM MgCl_2_, 10 mM KCl, 0.5 mM DTT, 0.5% Igepal, and EDTA-complete protease inhibitor cocktail) and incubated (15 min; on ice). After centrifugation (3500 rpm; 4 °C; 15 min), each supernatant, which contained predominantly cytoplasmic proteins, was collected. Each remaining pellet was washed two times and resuspended in nuclei-lysing buffer (5 mM HEPES pH 7.9, 1.5 mM MgCl_2_, 0.2 mM EDTA, 0.5 mM DTT, 25% glycerol and EDTA-free complete protease inhibitor cocktail) and incubated (4 °C; 25 min) with rocking agitation. Each supernatant, which contained predominantly total nuclear proteins, was collected after centrifugation (13,000 rpm; 4 °C; 5 min). Analysis of protein (20 μg) from cytoplasmic or nuclear cellular fractions was performed by western blot, as described above, using the antibodies anti-p-IRF3, anti-β-tubulin, and the polyclonal anti-Histone-3 (Cell Signaling), each at 1:1000 dilution.

### 2.5. Supernatant Collection and mRNA Extraction

MΦN or MΦP were cultured in 35-mm-diameter plastic Petri dishes (NUNC). Once 90% confluence was reached, each culture medium was replaced with RPMI containing 2% fetal bovine serum, to a final concentration of 1 × 10^6^ cells/mL. After the cultures were incubated (37 °C; 5% CO_2_) for the specified time (6, 24, or 48 h), supernatants were collected. Samples were frozen at −75 °C until use. After removing supernatants, total RNA from cells was extracted with the AxyPrep Multisource Total RNA Miniprep kit (Axygen Scientific, Union City, CA, USA), according to the manufacturer’s instructions.

### 2.6. Quantitation of IFN-β by ELISA

The concentration of IFN-β protein in MΦN and MΦP supernatants from 6, 24 and 48 h was determined by using a commercially available VeriKine™ mouse IFN-β sandwich ELISA kit (PBL interferon source, Piscataway, NJ, USA), according to the manufacturer’s instructions. From the IFN-β standard curve, samples with unknown concentration were interpolated. The assay range was 15.6–1000 pg/mL.

### 2.7. Quantitative RT-PCR

In a two-step RT-PCR assay, RNA (2 μg) was reverse transcribed with RNA transcriptase Superscript II (Invitrogen). TaqMan real-time RT-PCR was performed with primers and probes (Assay on Demand 20× mix) for murine *IFN-β, Mx1*, *ISG56* and *GAPDH* (endogenous control), using TaqMan assay reagent master mix (Applied Biosystems, Foster City, CA, USA). After determining the dynamic range, a cDNA dilution (1:25) was analyzed for expression of *IFN-β*, *Mx1*, *ISG56* and *GAPDH* genes. Cycling parameters were established according to the manufacturer’s protocol. Triplicate C_t_ values were analyzed by using the comparative C_t_ (ΔC_t_) method, as described by the manufacturer (Applied Biosystems). The relative amount of mRNA of each gene was obtained by normalization to the amount of mRNA of cellular *GAPDH*, as endogenous reference sample.

### 2.8. Macrophage Treatment with Recombinant IFN-β or Supernatants

Cultures of MΦN and MΦP, at 90% confluence, were each treated with 100 U/mL (26 ng/mL) of recombinant mouse-IFN-β (r-IFN-β) (Millipore Corporation, Temecula, CA, USA) (45 min; 37 °C; 5% CO_2_), as reported for RAW macrophages [39]. In addition, other MΦN were treated with supernatants collected from 24-h cultures of MΦP, previously exposed to U.V. light to inactivate the infectious RSV produced by MΦP and to eliminate the possibility of STAT1 activation by virus infection. Before U.V. light exposure, supernatants had a viral titer of 3.71 × 10^2^ ± 104 TCID_50_/mL; after treatment infectivity was null. Total RNA and whole cell-extracts were obtained as mentioned above and were analyzed by quantitative RT-PCR regarding *Mx1* and *ISG56* gene expression or by western blot with anti-STAT1, anti-p-STAT1 and anti-β-tubulin.

In order to analyze the activity of IFN-β from MΦP supernatants on STAT1 phosphorylation, IFN-α/β receptors expressed on MΦN were blocked with polyclonal mouse anti-IFNAR (R&D systems, Minneapolis, MN, USA) at a concentration of 10 μg/mL (30 min; 37 °C); thus treated, the MΦN were then incubated with the MΦP supernatants (45 min; 37 °C). Expression of p-STAT1 was evaluated by western blot.

### 2.9. Statistics

Two-sided, unpaired, Student’s *t*-test was applied to determine differences between groups, using GraphPad Prism 5.0 software (GraphPad, San Diego, CA, USA). A *p*-value of <0.05 was considered to indicate a statistically significant difference.

## 3. Results

### 3.1. Persistent RSV Infection is Maintained throughout Passages of MΦP

Persistent RSV-infection in the murine macrophage-like cell line P388D1 was monitored constantly by evaluating the expression of the viral structural proteins N and F and the expression of mRNA for the viral *N* gene. Flow cytometry showed that at least 96% of macrophages from passage 95 expressed RSV antigens (Supplementary Figure S1A) and the *N* gene mRNA was also expressed in passages 95 and 97 (Supplementary Figure S1B). These results were representative of the cellular passages used for this study, indicating that effectively, RSV is infecting persistently the MΦP culture.

### 3.2. IRF3 was Constitutively Expressed in Its Phosphorylated Form in MΦP

**Figure 1:**
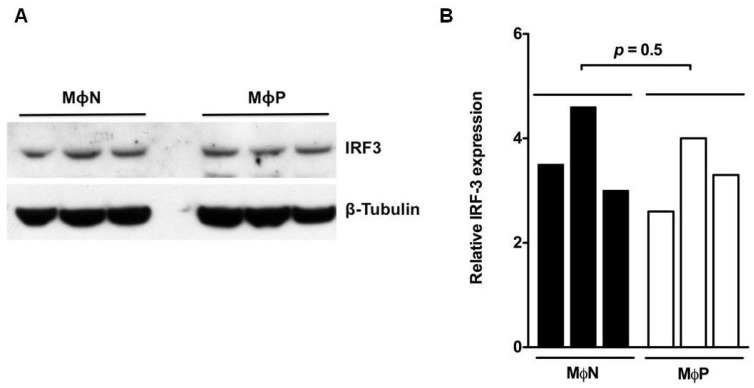
Total interferon regulatory factor 3 (IRF3) expression in persistently RSV-infected macrophages. (**A**) Total cell lysates from passages 86, 90 and 93 of non-infected macrophages (MΦN) and from passages 85, 86 and 88 of persistently RSV-infected macrophages (MΦP) were analyzed by western blot to detect total IRF3. The expression of β-tubulin protein was evaluated as loading control; (**B**) Semi-quantitative analysis was performed through densitometry with the software QuantityOne. Student-*t* test was performed to compare the average of the density values of the three passages of MΦN or MΦP. Results were reproduced in seven cellular passages analyzed (biological replicates).

The state of activation of the IFN-I signaling pathway during persistent RSV-infection was evaluated at the level of synthesis, by initial analysis of expression of the transcription factor IRF3. Western blot analysis (Figure 1A) showed that the levels of expression of total IRF3 in MΦP and MΦN were similar. Densitometry analysis (Figure 1B) showed no statistical difference between the average of three different passages from MΦN and MΦP (*p* = 0.5), indicating that constitutive expression of total IRF3 is not altered by persistent RSV-infection.

**Figure 2:**
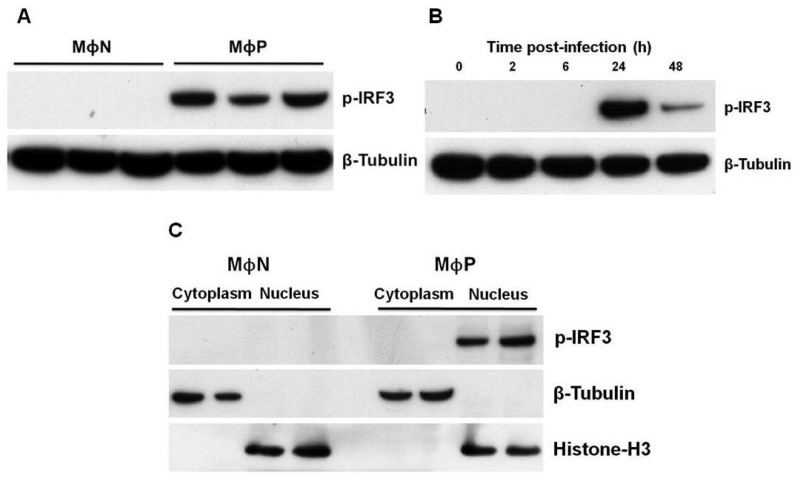
Phosphorylation and location of interferon regulatory factor 3 (IRF3) in persistently RSV-infected macrophages. (**A**) Phospho-IRF3 (p-IRF3) expression was analyzed by western blot in total cell lysates from passages 86, 90 and 93 of non-infected macrophages (MΦN) and from passages 85, 86 and 88 of persistently RSV-infected macrophages (MΦP); (**B**) As a positive control, induction of IRF3 phosphorylation was analyzed in acutely RSV-infected macrophages (m.o.i. of 2) at 2, 6, 24, and 48 h p.i. As loading control, the expression of β-tubulin was evaluated; (**C**) Cytoplasmic and nuclear subcellular fractions from passages 94 and 95 of MΦN and from passages 90 and 91 of MΦP were evaluated for localization of p-IRF3. To demonstrate the purity of the fractions, histone-H3 and β-tubulin (nuclear and cytoplasmic markers, respectively) were determined. Results were reproduced in six cellular passages analyzed (biological replicates).

During acute viral infections, including those by RSV, IRF3 is activated through serine/threonine phosphorylation, leading to its homo-dimerization and nuclear translocation [19]. Whether persistent RSV also changes the state of activation of IRF3 in MΦP, was analyzed through western blot. Figure 2A shows that MΦP express constitutively p-IRF3 while in MΦN this active form of IRF3 was not detected. As a control to verify that RSV infection is sufficient to induce IRF3 activation, MΦN were infected in acute manner (MΦA). It was observed in MΦA phosphorylation of IRF3 at 24 h p.i., (Figure 2B) with a significant reduction by 48 h p.i., confirming that RSV infection promotes activation of IRF3.

To determine the subcellular localization of p-IRF3, cells were fractionated into nuclear and cytoplasmic extracts. For MΦP, p-IRF3 was located exclusively in nuclei, whereas for MΦN, it was not detected in either cellular fraction, thus indicating that persistence of RSV induces constitutive phosphorylation of IRF3 and its translocation to the nuclei (Figure 2C). Then, p-IRF3 may be able to induce gene expression in a constitutive manner.

### 3.3. Persistent RSV Infection Induced Constitutive Expression of IFN-β

**Figure 3:**
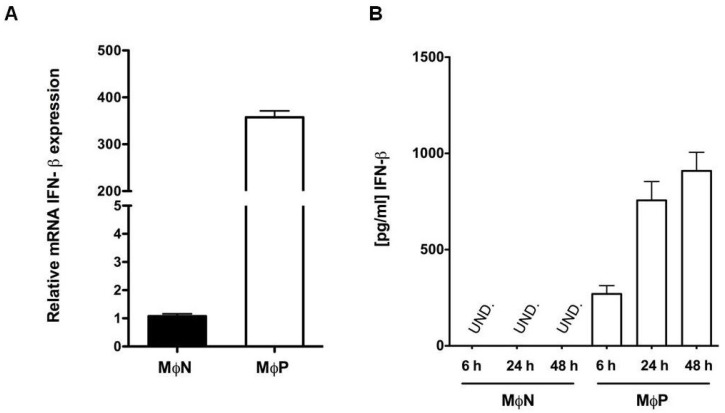
Expression of interferon-β (IFN-β) mRNA and protein in persistently RSV-infected macrophages. (**A**) Quantitative RT-PCR was performed with three different passages of non-infected macrophages (MΦN) and of persistently RSV-infected macrophages (MΦP), all cultured for 24 h, to determine the mRNA expression level of *IFN-β* by the ΔC_t_ method. The values from the three different passages were averaged and plotted as relative expression of mRNA *IFN-β*; (**B**) Quantitative analysis by ELISA of the expression of IFN-β protein in supernatants of MΦN and MΦP cultured for 6, 24, and 48 h. UND: undetected. IFN-β mRNA and protein expression were evaluated in passages 93, 95 and 96 of MΦN and 92, 93 and 94 of MΦP. Results were reproduced in six cellular passages analyzed (biological replicates).

To evaluate whether nuclear-p-IRF3 is active as transcription factor, it was determined the expression level of mRNA for *IFN-β*, as this gene is under control of IRF-3. MΦN and MΦP at 24 h of culture were analyzed by quantitative RT-PCR (qRT-PCR) by the ΔC_t_ method. Results showed that expression level of *IFN-β* mRNA in MΦP cultured for 24 h was more than 300-fold greater than that in MΦN (*p* < 0.0001) cultured in similar conditions (Figure 3A). No significant difference in *IFN-β* mRNA expression was observed between MΦP cultured for 6 and 24 h (351.6 ± 24.73- and 339.7 ± 22.07-fold increase, respectively in regard to MΦN). In order to determine whether *IFN-β* mRNA levels correlated with protein expression, supernatants from MΦP cultured for 6, 24, and 48 h were collected and analyzed by ELISA. Figure 3B shows a gradual increase in IFN-β expression over time, in the MΦP supernatants, possibly due to accumulation (270.1 ± 42.6, 756.20 ± 97.4, and 909.17 ± 96.6 pg/mL, respectively). No IFN-β was detected in the MΦN supernatants, despite their being cultured for 48 h. This result indicates that, although RSV persistence induces a constitutive production of IFN-β, MΦP are not able to eliminate the virus.

### 3.4. Persistent RSV Infection Impaired STAT1 Activation and ISGs Expression in Response to IFN-I

**Figure 4:**
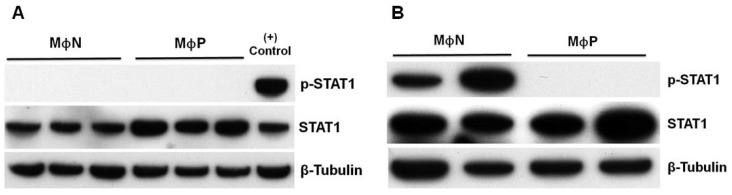
State of activation of transcription factor STAT1 in response to interferon-β (IFN-β) in persistently RSV-infected macrophages. (**A**) Total cell lysates of passages 95, 96 and 97 of non-infected macrophages (MΦN) and of passages 94, 95 and 96 of persistently RSV-infected macrophages (MΦP) were analyzed by western blot to detect total STAT1 and p-STAT1. As a positive control (+), MΦN were treated with 100 U/mL of recombinant-IFN-β for 45 min; (**B**) As MΦP do not respond to the IFN-β secreted by them, recombinant IFN-β (100 U/mL) was added to two passages of MΦP for 45 min. Two passages of MΦN were used as positive control. Results were reproduced in six cellular passages analyzed (biological replicates).

The activation of the IFN-I signaling pathway at the level of response was first evaluated in MΦP through the expression and activation of STAT1, a key player of the Jak/STAT signaling pathway, which is activated down-stream binding of IFNAR [40]. Total STAT1 was expressed constitutively in both MΦN and MΦP cultured for 48 h, but was slightly increased in MΦP (Figure 4A). However, p-STAT1 was not observed in any of the samples, although it had been expected that MΦP would have been positive for p-STAT1, because MΦP were constantly exposed to the IFN-β that they produced and secreted. A possible deficiency to signal in response to IFN-I by the macrophage-like cell line P388D1 was ruled out, because added commercial r-IFN-β induced phosphorylation of STAT1 in MΦN ((+) control, Figure 4A). In contrast, treatment of MΦP with a high concentration of r-IFN-β did not induce activation of STAT-1 (Figure 4B), confirming that activation of STAT1 during RSV persistence was impaired.

**Figure 5:**
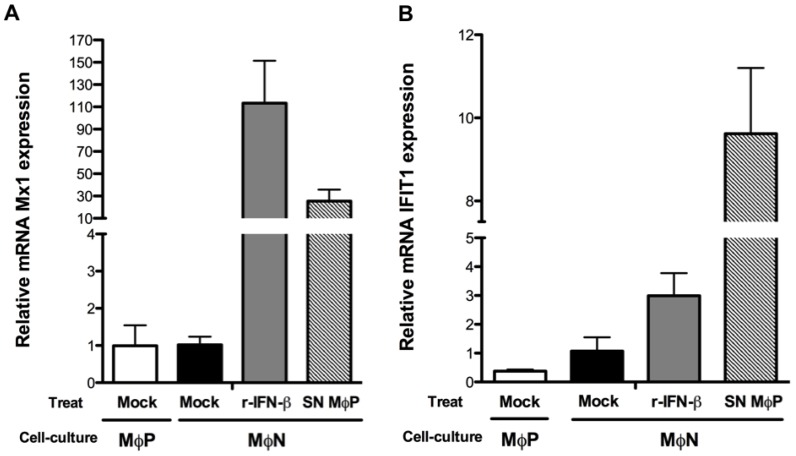
*Mx1* and *ISG56* mRNA expression level in persistently RSV-infected macrophages. Quantitative RT-PCR was performed with passages 107, 108 and 109 of mock-treated, non-infected macrophages (MΦN) and with passages 102, 103 and 104 of persistently RSV-infected macrophages (MΦP), to determine mRNA expression level of *Mx1* (**A**) and *ISG56* (**B**) by the ΔC_t_ method. Also, MΦN were treated with 100 U/mL of recombinant-IFN-β (r-IFN-β) or supernatants from passages 87, 90 and 96 of MΦP (SN MΦP) for 45 min. Subsequently, *Mx1* and *ISG56* gene expression was evaluated. The values from the three different passages were averaged and plotted as relative expression of each gene. Treat: treatment. Results were reproduced in six cellular passages analyzed (biological replicates).

A late consequence of the cellular response to IFN-I is the expression of antiviral genes [29]; thus the level of mRNA expression of the *Mx1* and *ISG56* genes in MΦP in respect to mock- or r-IFN-β-treated MΦN was then evaluated. Such genes have been previously reported as part of the early antiviral response during acute RSV infection [41,42]. As expected, relative expression of *Mx1* and *ISG56* mRNA in MΦP was similar to that in MΦN, notwithstanding constant exposure of MΦP to autocrine IFN-β (Figure 5A,B). Such result correlates with the lack of STAT1 phosphorylation in MΦP.

**Figure 6:**
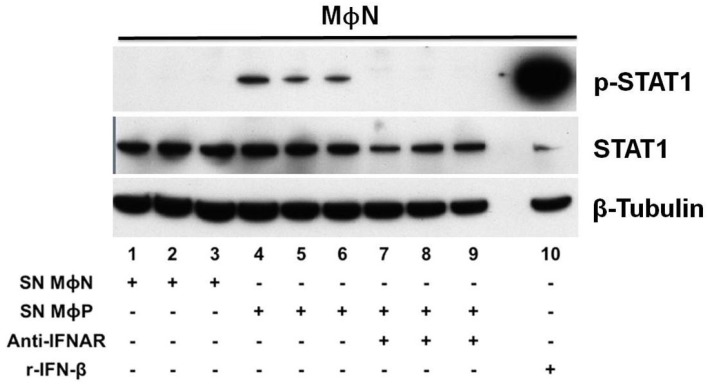
Activation of transcription factor STAT1 in non-infected macrophages in response to the interferon-β (IFN-β) produced by persistently RSV-infected macrophages. Three different passages of non-infected macrophages (MΦN) were incubated (45 min) with supernatants from three different passages of MΦN (lanes 1, 2, and 3) or with supernatants from passages 87, 90 and 96 of MΦP (lanes 4, 5, and 6), the latter supernatants containing MΦP-produced interferon-β (IFN-β). In addition, three passages of MΦN were pre-treated (30 min) with a polyclonal antibody against IFN α/β receptor (anti-IFNAR), at a concentration of 10 μg/mL, and then were incubated (45 min) with MΦP supernatants (lanes 7, 8, and 9). Total cell lysates were analyzed by western blot to detect total STAT1, p-STAT1, and β-tubulin as loading control. Infectious viruses present in the MΦP supernatants were inactivated by exposure to U.V. light to avoid viral infection as inducer of STAT1 activation. Treatment of a passage of MΦN with r-IFN-β (100 U/mL) was used as positive control (lane 10). Results were reproduced in six cellular passages analyzed (biological replicates).

To test whether the lack of autocrine response to IFN-β in MΦP was due to a deficient production of this cytokine or to impaired biological activity, three different passages of MΦN were treated with supernatants from MΦP cultures. In this case, STAT1 phosphorylation was induced in MΦN (Figure 6), along with the transcriptional activation of *Mx1* and *ISG56* genes (Figure 5A,B). Even more, when the IFN-I-receptor (IFNAR) in MΦN was blocked with a high concentration of a specific polyclonal antibody, subsequent exposition to supernatants from MΦP induced null levels of p-STAT1 (Figure 6). These findings indicate that the IFN-β produced by MΦP was of sufficient quantity and was capable of activating its specific signaling pathway in MΦN, but persistent RSV induced a refractory state to the IFN-I response in the host cell.

## 4. Discussion

Many viruses can establish and maintain persistent infections through interfering with, and controlling, antiviral cellular processes such as apoptosis and production of IFN-I [43]. Here, we report that RSV interfered with the IFN-I response during persistent infection in an *in vitro* model established in our laboratory, which consists of a murine macrophage cell line persistently infected with RSV (MΦP). The MΦP culture has been maintained over several passages, as confirmed through determining expression of viral *N* gene mRNA and the viral structural proteins N and F. Using this model, we observed that persistent RSV infection did not prevent either IRF3 activation or IFN-β expression in the host cell. However, the results indicate that persistent RSV infection inhibits the autocrine response to IFN-β down-stream binding the IFNAR, because neither STAT1 was phosphorylated nor *Mx1* and *ISG56* gene transcription were active in MΦP, even though we observed that this cytokine was constitutively produced and secreted into supernatants in sufficient quantities to activate MΦN. Moreover, MΦP were refractory to r-IFN-β stimuli, which induced STAT1 phosphorylation as well as antiviral genes expression in MΦN. Such results suggest that the lack of response to IFN-β by the persistently RSV-infected host cell may be one of several mechanisms for maintenance of RSV persistence.

Most studies conducted to analyze the consequences of RSV infection on IFN-I production and response have been performed by using models of acute infection. In comparison to other RNA viruses such as influenza and hMPV, RSV is considered as a low inducer of IFN-I due to the biological activity of the viral NS1 and NS2 proteins [44,45]. Epithelial cell lines and primary macrophages infected with mutant RSV lacking the NS1/NS2 genes produce higher levels of IFN-I than when infected with wtRSV [31,34,46]. Particularly, it has been reported that wtRSV infection of A546 cells induces activation and nuclear localization of IRF3; however, detection of this transcription factor is dramatically diminished with the onset of NS1/NS2 expression [34], due mainly to protein degradation [47]. Comparison between MΦP and MΦN showed no difference in total IRF3 expression, but the phosphorylated form was detected only in RSV-infected cultures. For MΦA, a high level of p-IRF3 was detected at 24 h p.i., with an important reduction by 48 h p.i., possibly associated with the expression of NS1/NS2. In contrast, that p-IRF3 was expressed in MΦP at similar levels after 24 or 48 h (data not shown), suggests equilibrium between production of viral and cellular proteins in the host cell during persistent RSV infection.

Nuclear localization of p-IRF3 correlated with the constitutive expression of mRNA of *IFN-β* gene and the secreted protein, indicating that its activation and role as a transcription factor is not impaired in macrophages by RSV persistence. To our knowledge, there is only one study directed to evaluate the state of activation of the IFN-I signaling pathway during RSV persistence and it was performed in in Hep-2 cells [48]. In agreement with our observations in kinetic assays to quantify the IFN-β produced at 6, 24 and 48 h by MΦP (shown in Figure 3B), it was reported that persistent RSV infection in Hep-2 cells also induces IFN-β expression, although it was quantified after 8 h of culture [48]. Expression of other genes directly controlled by IRF3 was not analyzed in our *in vitro* model, however we have observed high production of the chemokine RANTES [49], which is product of a gene that requires cooperation between IRF3 and NF-kB [50]. Such observations suggest that a subset of genes under the control of IRF3 may be constitutively expressed in MΦP.

We found the continuous production of IFN-β in MΦP is paradoxical, because these cells do not eliminate the persistent RSV. This finding may be explained by the lack of autocrine response to the constitutively produced IFN-β. This deficiency in response to IFN-I is in agreement with previous reports in which acute RSV infection in primary dendritic cells and RAW macrophages was shown to induce expression of total STAT1 and STAT2, whereas their phosphorylated forms were not found—Even after r-IFN-β had been added [39,51]. In contrast to reports of epithelial cells in which STAT2 is degraded by the expression of NS2 or during RSV infection [35,47,52], it appears that inhibition of response to IFN-I in myeloid cells is mediated by increased phosphatase activity, preventing Tyk2 phosphorylation with a subsequent deficiency in STAT2 and STAT1 activation, rather than degradation [39]. Otherwise, overexpression of suppressor of cytokine signaling 1 (SOCS1) during persistent RSV infection in Hep-2 cells is also a possible mechanism responsible for inhibition of STAT1/STAT2 phosphorylation [48]. In MΦP, we observed a slightly higher expression of total STAT1, than that in MΦN, but p-STAT1 was not detected after addition of r-IFN-β at a concentration that had been sufficient to activate STAT1 in MΦN. These results suggest that persistent RSV more efficiently controls the response to IFN-I than its production, by inhibition of STAT1 phosphorylation with the concomitant lack of ISGs transcription, allowing viral persistence. The biological activity of the IFN-β produced and secreted by MΦP was evidenced, because induced STAT1 phosphorylation and ISGs expression in MΦN and their activation was inhibited blocking the IFNAR with a specific polyclonal antibody. In future investigations, we will study the mechanism involved in the impairment of the IFN-I response in MΦP. Importantly, the paracrine effect of supernatants from MΦP on MΦN may lead to the acquisition of a proinflammatory phenotype, since antiviral response was activated in non-infected macrophages. In fact, supernatants from MΦP were more efficient than r-IFN-β to induce expression of the *ISG56* gene (shown in Figure 5B).

Studying the infectious process by RSV in macrophages is relevant since during severe acute lower-respiratory tract disease in children, analysis of bronchioalveolar lavages has shown that alveolar macrophages are target cells for this virus, because they express RSV antigens and proinflammatory cytokines [53]. Also, it has been reported that isolated human alveolar macrophages can support a productive RSV infection *in vitro* at least for 25 days without a notable reduction in viability [54], suggesting that macrophages may be an important reservoir for RSV during acute infection *in vivo* and possibly during persistent infection, although this type of infection has not been confirmed in humans.

## 5. Conclusions

Overall, our work extends the information about the state of activation of the IFN-I signaling pathway during persistent RSV infection in macrophages, through three meaningful findings: 1) a constitutive state of activation of IRF3 resulting in a continual IFN-β production and secretion; 2) the lack of autocrine response to the constitutively produced IFN-β by inhibition of STAT1 phosphorylation, preventing transcription of antiviral genes and consequently allowing maintenance of persistent RSV infection; 3) the paracrine effect of the constitutively produced IFN-β, on non-infected macrophages inducing STAT1 activation and transcription of antiviral genes. We hypothesized that the paracrine effect of the IFN-I expressed by persistently infected macrophages, on non-infected cells may have relevant implications for the development of a chronic proinflammatory environment contributing to the pathogenesis of RSV infection.

## Supplementary Files

Supplementary File 1
