# Supplementary material for: Respiratory Syncytial Virus Persistence in Murine Macrophages Impairs IFN-β Response but Not Synthesis
Source: Viruses. 2015 Oct 16;7(10):5361–74. doi: 10.3390/v7102879 (PMC4632387; doi:10.3390/v7102879)
Supplement: Supplementary File 1 [file viruses-07-02879-s001.pdf]

Supplementary Information

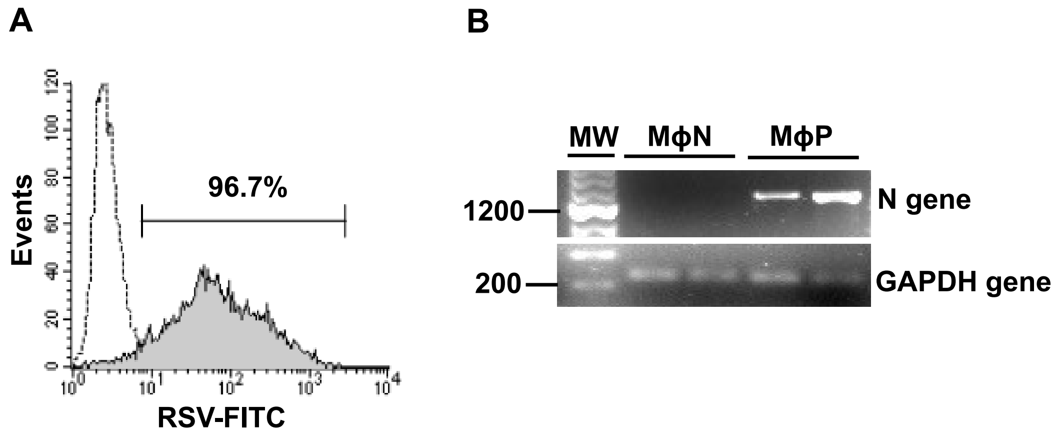

Figure S1. Persistent infection in murine macrophages. (**A**) Direct immunofluorescence to detect structural viral proteins by flow cytometry. Persistently RSV-infected macrophages (MΦP) from passage number 95 (solid line) and non-infected macrophages (MΦN) from passage 85 (dashed line) were stained under the same conditions to detect RSV proteins. Percentage of positive cells is reported; (**B**) Conventional RT-PCR to detect the expression of *N* gene. Two different passages of MΦP (95 and 97) were analyzed, along with two passages of MΦN (P85 and P90) as negative control. Expression of *GAPDH* gene was analyzed as endogenous control.
